# Generative Models Should at Least Be Able to Design Molecules That Dock Well: A New Benchmark

**DOI:** 10.1021/acs.jcim.2c01355

**Published:** 2023-05-24

**Authors:** Tobiasz Ciepliński, Tomasz Danel, Sabina Podlewska, Stanisław Jastrzȩbski

**Affiliations:** † Faculty of Mathematics and Computer Science, Jagiellonian University, Łojasiewicza 6, 30-348 Kraków, Poland; ‡ Maj Institute of Pharmacology, Polish Academy of Sciences, Smȩtna 12, 31-343 Kraków, Poland; ¶ Molecule.one, Al. Jerozolimskie 96, 00-807 Warsaw, Poland

## Abstract

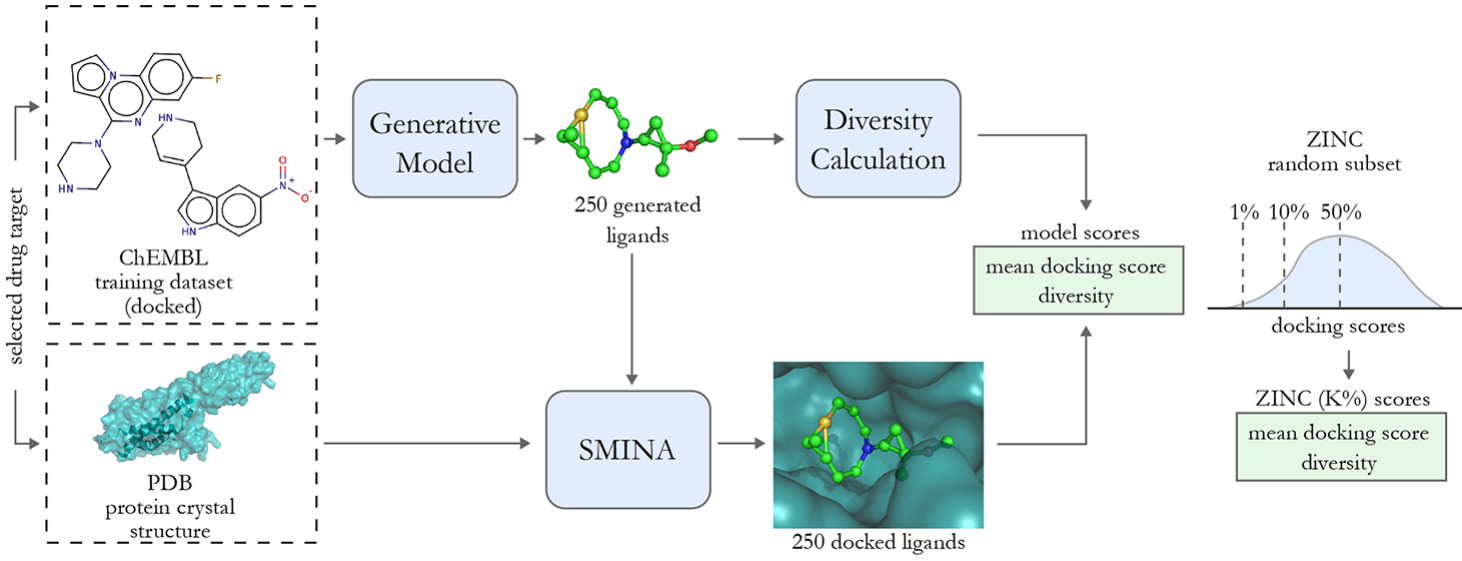

Designing compounds with desired properties is a key element of the drug discovery process. However, measuring progress in the field has been challenging due to the lack of realistic retrospective benchmarks, and the large cost of prospective validation. To close this gap, we propose a benchmark based on docking, a widely used computational method for assessing molecule binding to a protein. Concretely, the goal is to generate drug-like molecules that are scored highly by SMINA, a popular docking software. We observe that various graph-based generative models fail to propose molecules with a high docking score when trained using a realistically sized training set. This suggests a limitation of the current incarnation of models for *de novo* drug design. Finally, we also include simpler tasks in the benchmark based on a simpler scoring function. We release the benchmark as an easy to use package available at https://github.com/cieplinski-tobiasz/smina-docking-benchmark. We hope that our benchmark will serve as a stepping stone toward the goal of automatically generating promising drug candidates.

## Introduction

Designing compounds with some desired chemical properties is the central challenge in the drug discovery process.^1,2^*De novo* drug design is one of the most successful computational approaches that involves generating new potential ligands *from scratch*, which avoids enumerating explicitly the vast space of possible structures. Recently, deep learning has unlocked new progress in drug design. Promising results using deep generative models have been shown in generating soluble,^3^ bioactive,^4^ and drug-like^5^ molecules. The history of *de novo* compound design dates back to the 1980s.^6^ Since then, numerous other approaches emerged, from both ligand- and structure-based path.^7,8^ Despite existing cheminformatic approaches to new compounds generation, it was the introduction of machine learning (ML) into the field of the computer-aided drug design that revolutionized also the task of *de novo* ligand design. In recent years, the combination of ML with the information on the target is gaining significant popularity.

A key challenge in the field of drug design is the lack of realistic benchmarks.^2^ Ideally, the generated molecule by a *de novo* method should be tested in the wet lab for the desired property. In practice, typically, a proxy is used. For example, the octanol–water partition coefficient or bioactivity is predicted using a computational model.^3,4^ However, these models are often too simplistic.^2^ This is aptly summarized by Coley et al.^10^ who notice that the current generative model benchmarks fail to capture the complexity of real discovery problems. In contrast to drug design, more realistic benchmarks have been used in the design of photovoltaics^11^ or in the design of molecules with certain excitation energies,^12^ where a physical calculation was carried out both to train models and to evaluate generated compounds. A huge step toward unifying chemical benchmark was made by Huang et al.^13^ who introduced an open-source benchmark, Therapeutics Data Commons, and showed that current algorithms are yet not primed to solve all the key therapeutic challenges. Despite this, the recent advances in deep learning have already led to numerous successful applications in drug discovery projects.^14^

Recently, an increasing number of methods adopts molecular docking as a means of evaluation for generative models in drug design.^15−17^ More specifically, in computer-aided drug discovery pipelines, docking scores are often used to preliminarily assess proposed drug candidates before reaching for costly laboratory experiments.^18−20^ With an advent of geometric deep learning for molecular graphs, the structure-based generative models, often employing roto-translationally equivariant neural networks, began to develop rapidly.^21−24^ Many of these methods use molecular docking to guide the generative process, so the docking scores are the most natural way of the compound evaluation.

**Figure 1:**
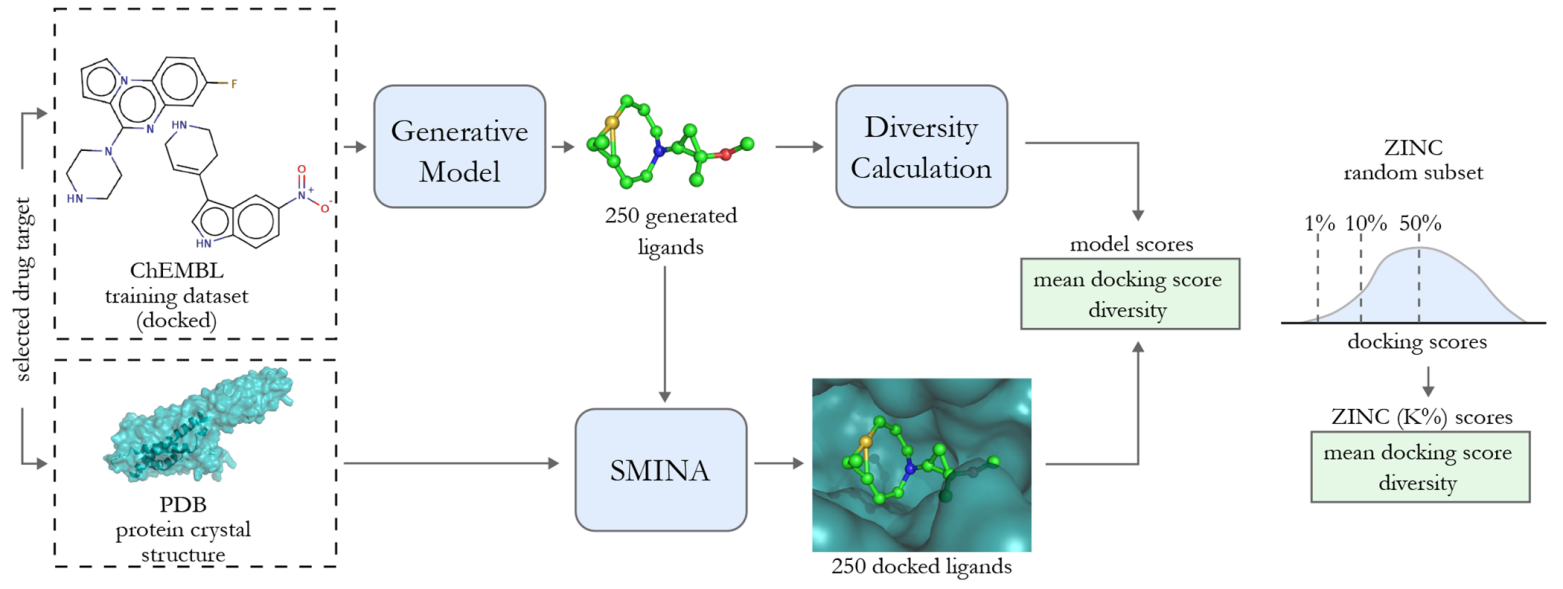
Visualization of the proposed docking-based benchmark for *de novo* drug design methods. First, the generative model is trained for a selected drug target and generates 250 ligand proposals. The model score is a combination of the mean docking score (or single docking score component, e.g., repulsion or hydrogen bonding) of the generated compounds and their diversity. As a reference value, we use the scores of the top K% of a random ZINC subset (depicted on the right side).

Our main contribution is a realistic benchmark for *de novo* drug design (Figure 1). We base our benchmark on docking, a popular computational method for predicting molecule binding to a protein. Concretely, the goal is to generate molecules that are scored highly by SMINA.^25^ We picked Koes et al.^25^ due to its popularity and being available under a free license. While we focus on *de novo* drug design, our methodology can be extended to evaluate retrospectively other approaches to designing molecules. Code to reproduce results and evaluate new models is available online at https://github.com/cieplinski-tobiasz/smina-docking-benchmark. Notably, our benchmark^26^ was already adopted by Nigam et al.^27^ to demonstrate the effectiveness of their genetic algorithm for molecular design.

Our second contribution is exposing the limitation of currently popular *de novo* drug design methods for generating bioactive molecules. When trained using a few thousands compounds, a typical training set size, the tested methods fail to generate highly active structures according to the docking software. The highest scoring molecules in most cases did not outperform the top 10% molecules found in either the ZINC database or the training set. This suggests we should exercise caution when applying them in drug discovery pipelines, where we seldom have a larger number of known ligands. We hope our benchmark will serve as a stepping stone to further improve these promising models.

The paper is organized as follows. We first discuss prior work and introduce our benchmark. Next, we use our benchmark to evaluate two popular models for *de novo* drug design. Finally, we analyze why the tested models fail on the most difficult version of the benchmark.

## Docking-Based Benchmark

We begin by briefly discussing prior work and motivation. Next, we introduce our benchmark.

### Why Do We Need Yet Another Benchmark?

Standardized benchmarks are critical to measure progress in any field. Development of large-scale benchmarks such as the ImageNet was critical for the recent developments in artificial intelligence.^28,29^ Many new methods for *de novo* drug design are conceived every year, which motivates the need for a systematic and efficient way to compare them.^30^

*De novo* drug design methods are typically evaluated using *proxy tasks* that circumvent the need to test the generated compounds experimentally.^3,5,31−33^ Optimizing the octanol–water partition coefficient (log *P*) is a common example. The log *P* coefficient is commonly computed using an atom-based method that involves summing contribution of individual atoms,^5,34^ which is available in the RDKit package.^35^ Due to the fact that it is easy to optimize the atom-based method by producing unrealistic molecules,^36^ a version that heuristically penalizes hard to synthesize compounds is used in practice.^5^ This example illustrates the need to develop more realistic ways to benchmark these methods. Another example is QED score,^37^ which is designed to capture the *drug likeliness* of a compound. Finally, some approaches use a model (e.g., a neural network) to predict bioactivity of the generated compounds.^4^ Similarly to log *P*, these two tasks are also possible to optimize while producing unrealistic molecules. This is aptly summarized in Coley et al.^10^ as

“The current evaluations for generative models do not reflect the complexity of real discovery problems.”

Interestingly, besides the aforementioned proxy tasks, more realistic proxy tasks are rarely used in the context of evaluating *de novo* drug design methods. This is in contrast to evaluation of generative models for generating photovoltaics^11^ or molecules with certain excitation energies.^12^ One notable exception is Aumentado-Armstrong^38^ who try to generate compounds that are active according to the DrugScore^39^ and then evaluate the generated compounds using rDock.^40^ This lack of the overall diversity and realism in the typically used evaluation methods motivates us to propose our benchmark, which uses molecular docking as a more realistic proxy task.

Arguably, docking-based scoring of compounds has serious limitations,^41^ and similarity-based models^36^ are often chosen in commercial projects over molecular docking.^42^ However, the idea of our benchmark is that docking, even if simplistic, proves to be challenging for generative models. Our setup aims to imitate real drug discovery scenarios by employing this simple docking proxy.

### Docking-Based Benchmark

Our docking-based benchmark is defined by (1) docking software that computes for a generated compound its pose in the binding site, (2) a function that scores the pose, and (3) a training set of compounds with an already computed docking score.

The goal is to generate 250 molecules that achieve the maximum possible docking score. We find this number of compounds large enough to make the optimization of diversity nontrivial, but small enough to make testing feasible in practice (in terms of either computational resources or the cost of ordering compounds for wet lab experiments). For the sake of simplicity, we do not impose limits on the distance of the proposed compounds to the training set. Thus, a simple baseline is to return the training set. Finding similar compounds that have a higher docking score is already prohibitively challenging for current state-of-the-art methods. As the field progresses, our benchmark can be easily extended to account for the similarity between the generated compounds and the training set.

Finally, we would like to stress that the benchmark is not limited to *de novo* methods. The benchmark is applicable to any other approaches such as virtual screening. The only limitation required for a fair comparison is that docking is performed only on the supplied training set.

### Instantiation

As a concrete instantiation of our docking-based benchmark, we use SMINA v. 2017.11.9^25^ due to its widespread use and its being offered under a free license. To create the training set, we download from the ChEMBL^43^ database molecules tested against selected drug targets: 5-HT1B, 5-HT2B, ACM2, and CYP2D6. In the extended variant of our benchmark, we include four additional drug targets: ADRB1, MOR, A2A, and D2. For instance, the final 5-HT1B data set consists in 1,878 molecules, out of which 1,139 are active (*K*_i_ < 100 nM) and 739 are inactive molecules (*K*_i_ > 1,000 nM). Only molecules that dock successfully are retained. We list the resulting data set sizes in Table 1.

**Table 1:** Sizes of the Data Set Used in the Benchmark^a^

	5HT1B	5HT2B	ACM2	CYP2D6	ADRB1	MOR	A2A	D2
Data set size	1878	1193	2337	4199	1082	10225	9326	9509
# Actives	1139	656	1300	343	86	1094	1084	419
# Inactives	739	537	1037	3856	996	9131	8242	9090

a The corresponding test data set comprises of 10% of the whole data set, and the rest of it is used in training.

We dock each molecule using default settings in SMINA to a manually selected binding site coordinate. Protein structures were downloaded from the Protein Data Bank, cleaned and prepared for docking using Schrödinger modeling package. The resulting protein structures are provided in our code repository. We describe further details on the preparation of the data sets in the Supporting Information.

Starting from the above, we define the following three variants of the benchmark. In the first variant (Docking Score Function), the goal is to propose molecules that achieve the smallest Vinardo docking score^44^ (based on the Vina docking score^45^) used in the score_only mode of the SMINA package, defined as follows:
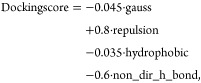
where all terms are computed based on the final docking pose. The first three terms measure the steric interaction between ligand and the protein. The fourth and the fifth terms look for hydrophobic and hydrogen bonds between the ligand and the protein. We include a detailed description of all the terms in the Supporting Information.

Next, we propose two simpler variants of the benchmark based on individual terms in the Vinardo scoring function. We select optimization targets that have clear interpretations: repulsion that minimizes clashes with protein and hydrogen bonding that maximizes interactions stabilizing the compound pose in the binding site. In the Repulsion task, the goal is to only minimize the repulsion component, which is defined as
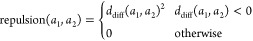
where *d*_diff_(*a*_1_, *a*_2_) is the distance between the atoms minus the sum of their van der Waals radii. The distance unit is Angstrom (10^–10^ m).

The third task, Hydrogen Bonding, is to maximize the non_dir_h_bond term:
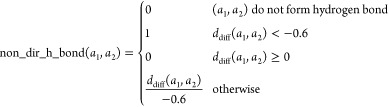


To make the results more stable, we average the score over the top 5 best-scoring binding poses. Finally, to make the benchmark more realistic, we filter the generated compounds using the Lipinski rule and discard molecules with molecular weights lower than 100.

## ZINC Baseline

The premise behind *de novo* drug discovery is that it enables access to structurally novel and potent molecules. To contextualize results in the benchmark, we included as the baseline sampling from the subset of ZINC database containing 9,204,719 molecules.^46^ We selected molecules having the following properties: 3D representation, standard reactivity, in-stock purchasability, ref pH, charges from −2 to +2 inclusive, and a used drug-like preferred subset. For each protein we have sampled a set of molecules from aforementioned ZINC subset of the protein’s training set size. In each task, we compare to the mean value of the top 50%, 10%, and 1% of scores.

### Diversity

**Figure 2:**
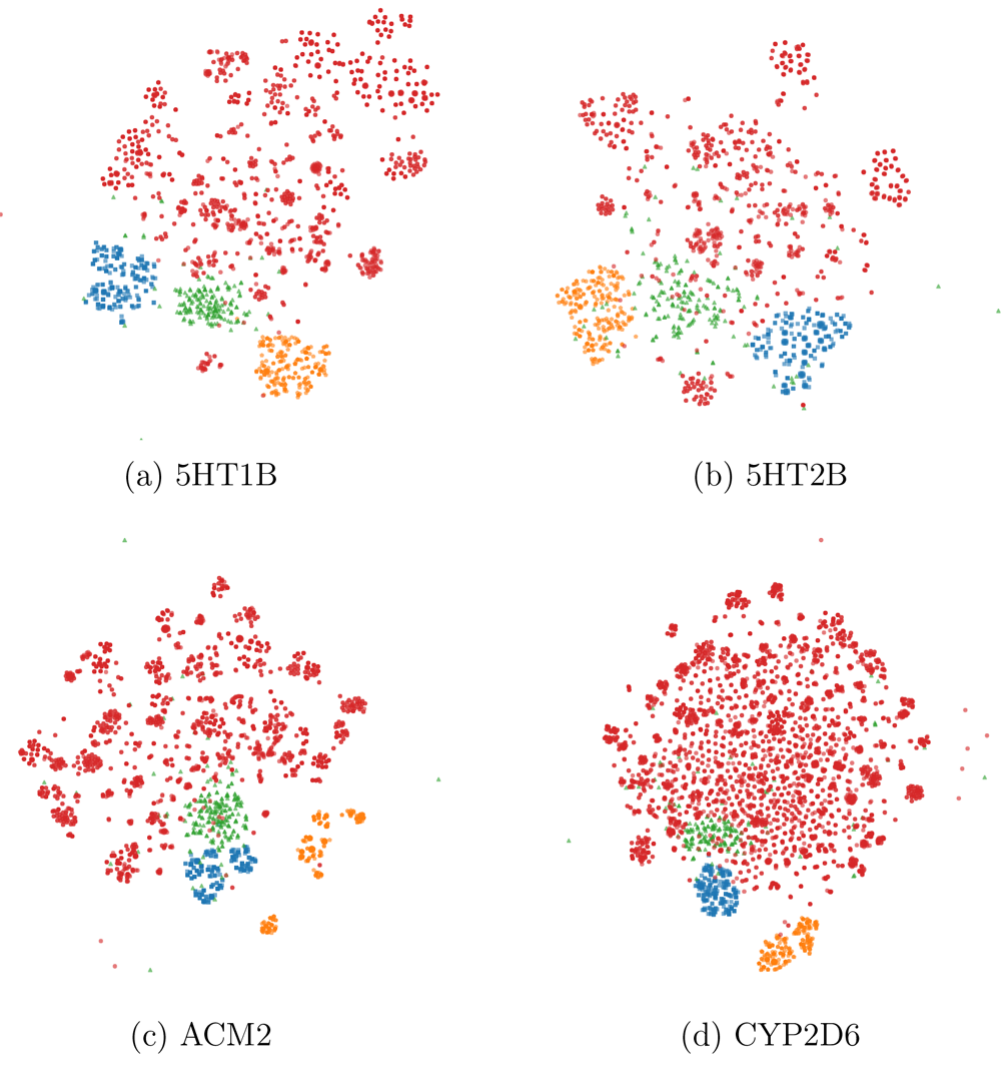
t-SNE maps of compound fingerprints (ECFP) for each protein. Each point is a molecule, and the distances between points are proportional to the dissimilarity of compounds. The training set is marked with red dots, and the compounds generated by REINVENT by enhancing different optimization targets are colored in blue (Docking Score Function), orange (Hydrogen Bonding), and green (Repulsion).

To better understand the performance of each model, besides the mean score, we also evaluate the diversity of the proposed molecules. Concretely, we compute the mean Tanimoto distance between all pairs of molecules in the generated sample. We use the 1024-bit ECFP representation^47^ with radius 2. The diversity score is reported in the benchmark along with the docking score results. We observe that the optimized models narrow down to a less diverse subspace of compounds that are dissimilar to the training set. This can also be observed in the t-SNE plots of the generated compounds compared to the training set (Figure 2). In this figure, compounds are grouped together based on their structural similarity. The small focused clouds of compounds generated using different optimization targets always concentrate at one side of the map. This suggests that there is similar bias of the model independent of the optimization target, which can be the ChEMBL prior to the REINVENT model^48^ (described below as one of the compared generative models). The separation between the optimized compounds and the training set suggests that these are novel molecules (similar structures are rarely present in the training set). Besides that observation, we note that the generated compounds are less diverse, creating one dense blob instead of multiple clusters, where all compounds are similar to each other inside one optimization target.

### When Is a Task Solved?

In the experiments, we compare to two baselines: (i) random compounds from ZINC as the baseline and (ii) compounds from the training set. In each case we report the mean score, the top 10% of scores, and the top 1% of scores. We also report diversity of the results.

Roughly speaking, we consider a given optimization task solved by a generative model if the molecules generated by this model achieve a mean score that exceeds the score of top 1% compounds in the ZINC subset (the values provided in theResultssection), while achieving at least the same diversity as observed in the training set of activity data extracted from ChEMBL (also provided below for each protein). This criterion is necessarily arbitrary. It is inspired by a natural baseline—comparing against a random sample of several thousands of drug-like compounds from the ZINC database.

### Model Evaluation Workflow

Below, we summarize all the steps necessary to evaluate a generative model and compare it with our benchmark. A general overview of this workflow is depicted in Figure 1, and the step-by-step evaluation procedure is shared in our code repository in the Python notebook named getting-started.ipynb.

1. Download the activity data associated with the selected drug target using the link provided in our code repository. This data contain both activity classes (active or inactive based on the experimental *K*_i_) and docking scores.
2. Use the provided data to train a generative model that optimizes docking scores (or other optimization target) and generate 250 unique compounds.
3. The generated compounds should be filtered using the Lipinski rule, and each molecule should have molecular weights greater than 100.
4. Dock the filtered set of compounds and calculate its diversity and the mean value of the optimization target.
5. Repeat for all proteins in the benchmark and all optimization targets.

## Results and Discussion

In this section, we evaluate three popular models for *de novo* drug design on our docking-based benchmark.

### Models

We compare three popular models for *de novo* drug design. Chemical Variational Autoencoder (CVAE)^49^ applies Variational Autoencoder^50^ by representing molecules as strings of characters (using SMILES encoding). This approach was later extended by Grammar Variational Autoencoder (GVAE),^3^ which ensures that generated compounds are grammatically correct. The third model, REINVENT,^48^ is a recurrent neural network that generates SMILES strings. It is first trained in a supervised manner to produce correct drug-like compounds similar to the ChEMBL data set (prior). Next, it is trained using reinforcement learning to optimize docking scores, which are provided as a training reward.

We note that CVAE and GVAE were not designed for small sample sizes, so they may not fully exploit their potential in our benchmark since they are used out of context. On the other hand, it was shown that REINVENT is a representative method in terms of achieving high sample efficiency.^51^

### Experimental Details

To generate active compounds, we follow an approach similar to the one in Jin et al.,^5^ disregarding the penalty for insufficient similarity. Analogous methods using the sparse Gaussian Process instead of a multilayer perceptron are also employed in Gómez-Bombarelli et al.^49^ and Kusner et al.^3^ The exact algorithm for training our generative models is described below.

First, we fine-tune a given generative model for 5 epochs on the training set ligands, starting from weights made available by the authors. All hyperparameters are set to default values used in Gómez-Bombarelli et al.^49^ and Kusner et al.^3^ Additionally, we use the provided scores to train a multilayer perceptron (MLP) to predict the optimization target (e.g., the SMINA scoring function) based on the latent space representation of the molecule.

For CVAE and GVAE, to generate compounds, we first take a random sample from the latent space by sampling from a Gaussian distribution with the standard deviation of 1 and the mean of 0. Starting from this point in the latent space, we take 50 gradient steps to optimize the output of the MLP. Based on this approach we generate 250 compounds from the model.

For the REINVENT model, we use pretrained weights on the ChEMBL database provided by Olivecrona et al.^48^ As there is no latent space in this model, we train a random forest model to predict the optimization target directly from the molecule structure. We use the ECFP fingerprint to encode the molecule.^47^ The reward is computed based on the random forest prediction multiplied by the QED score calculated using RDKit.

The key limitation of all the generative methods above is the use of an ML model, either an MLP or a random forest, to predict docking scores. This is an important design decision in this study. Our benchmark aims to simulate a setting in which a drug discovery campaign involves designing a small batch of compounds to be tested for biological activity based on prior biological data. This is different from searching for a highly docking compound, in which case a reasonable approach would be to compute docking scores for a large number of test compounds.

All other experimental details, including hyperparameter values used in the experiments, can be found in the appendix.

### Optimization of Docking Objectives

Tables 2 and 3 summarize the results on all three tasks. Recall that we generally consider a given task solved if the generated molecules exceed the top 1% score found in the ZINC database, while achieving at least the same diversity as in the training set. Below we make several observations.

**Table 2:** Results on the Three Molecule Generation Tasks, Each Rerun for Four Different Proteins, Composing Our Docking-Based Benchmark^a^

	5HT1B		5HT2B		ACM2		CYP2D6	
(a) Docking Score Function (*↓*)								
CVAE	–4.647	(0.907)	–4.188	(0.913)	–4.836	(0.905)	-	-
GVAE	–4.955	(0.901)	–4.641	(0.887)	–5.422	(0.898)	-	-
REINVENT	**-9.774**	(0.506)	**-8.657**	(0.455)	**-9.775**	(0.467)	**-8.759**	(0.626)
Train (50%)	–8.541	(0.850)	–7.709	(0.878)	–6.983	(0.868)	–6.492	(0.897)
Train (10%)	–10.837	(0.749)	–9.769	(0.831)	–8.976	(0.812)	–9.256	(0.869)
Train (1%)	–11.493	(0.859)	–10.023	(0.746)	–10.003	(0.773)	–10.131	(0.763)
ZINC (50%)	–7.886	(0.884)	–7.350	(0.879)	–6.793	(0.873)	–6.240	(0.883)
ZINC (10%)	–9.894	(0.862)	–9.228	(0.851)	–8.282	(0.860)	–8.787	(0.853)
ZINC (1%)	–10.496	(0.861)	–9.833	(0.838)	–8.802	(0.840)	–9.291	(0.894)
(b) Repulsion (*↓*)								
CVAE	**1.148**	(0.919)	**1.001**	(0.914)	**1.132**	(0.908)	**2.234**	(0.914)
GVAE	1.361	(0.910)	1.159	(0.942)	1.383	(0.917)	-	-
REINVENT	1.544	(0.811)	1.874	(0.859)	2.262	(0.845)	2.993	(0.858)
Train (50%)	2.099	(0.845)	1.792	(0.881)	1.434	(0.863)	6.508	(0.895)
Train (10%)	0.835	(0.863)	0.902	(0.893)	0.779	(0.888)	2.823	(0.904)
Train (1%)	0.550	(0.858)	0.621	(0.963)	0.553	(0.921)	1.284	(0.956)
ZINC (50%)	1.803	(0.878)	1.677	(0.882)	1.665	(0.879)	5.786	(0.880)
ZINC (10%)	0.840	(0.880)	0.865	(0.896)	0.792	(0.881)	2.348	(0.887)
ZINC (1%)	0.613	(0.941)	0.625	(0.922)	0.612	(0.938)	1.821	(0.880)
(c) Hydrogen Bonding (*↑*)								
CVAE	1.089	(0.915)	1.168	(0.909)	0.881	(0.907)	0.539	(0.908)
GVAE	**4.152**	(0.921)	**2.954**	(0.912)	2.567	(0.927)	**2.732**	(0.902)
REINVENT	3.795	(0.626)	2.451	(0.580)	**3.520**	(0.480)	1.304	(0.574)
Train (50%)	1.069	(0.843)	0.668	(0.882)	0.296	(0.871)	0.684	(0.892)
Train (10%)	2.934	(0.751)	2.327	(0.816)	1.444	(0.896)	2.061	(0.884)
Train (1%)	3.351	(0.825)	3.586	(0.575)	2.519	(0.852)	2.700	(0.917)
ZINC (50%)	1.114	(0.879)	0.871	(0.882)	0.512	(0.877)	0.660	(0.877)
ZINC (10%)	3.623	(0.873)	2.674	(0.887)	2.449	(0.874)	1.831	(0.878)
ZINC (1%)	5.743	(0.928)	3.545	(0.935)	3.253	(0.940)	2.115	(0.861)

a The key task is Docking Score Function in which the goal is to optimize the docking score against a given drug target. Each cell reports the mean score for 250 generated molecules in each task. In the parentheses, the internal diversity of generated molecules is reported (see text for details). The tested models tend to improve upon the mean score in the ZINC database (ZINC). However, they generally do not improve upon the top molecules from ZINC; ZINC (10%) and ZINC (1%) show the top 10% of scores and the top 1% of scores. Missing results (“-”) indicate that the model failed to generate 250 molecules that satisfy drug-like filters (see text for details).

**Table 3:** Results on the Three Molecule Generation Tasks for the Four Additional Drug Targets^a^

	ADRB1		MOR		A2A		D2	
(a) Docking Score Function (*↓*)								
CVAE	–4.581	(0.920)	–4.962	(0.911)	–4.545	(0.917)	–5.151	(0.913)
GVAE	-	-	-	-	-	-	-	-
REINVENT	**-8.164**	(0.831)	**-7.326**	(0.821)	**-7.372**	(0.821)	**-8.265**	(0.815)
Train (50%)	–12.084	(0.712)	–8.340	(0.837)	–7.725	(0.846)	–10.118	(0.829)
Train (10%)	–13.246	(0.534)	–9.174	(0.843)	–8.617	(0.858)	–11.451	(0.828)
Train (1%)	–13.929	(0.400)	–9.959	(0.828)	–9.839	(0.853)	–12.416	(0.769)
ZINC (50%)	–9.189	(0.866)	–8.046	(0.870)	–7.755	(0.874)	–9.094	(0.869)
ZINC (10%)	–10.361	(0.852)	–8.959	(0.863)	–8.807	(0.869)	–10.341	(0.859)
ZINC (1%)	–11.299	(0.844)	–9.808	(0.857)	–9.778	(0.869)	–11.424	(0.847)
(b) Repulsion (*↓*)								
CVAE	**1.188**	(0.916)	**1.704**	(0.912)	**0.898**	(0.911)	**1.648**	(0.914)
GVAE	1.433	(0.931)	-	-	-	-	-	-
REINVENT	2.370	(0.867)	2.163	(0.876)	2.355	(0.839)	2.225	(0.858)
Train (50%)	2.906	(0.834)	2.175	(0.851)	1.028	(0.826)	1.842	(0.850)
Train (10%)	1.656	(0.857)	1.301	(0.859)	0.804	(0.826)	1.214	(0.856)
Train (1%)	0.855	(0.829)	0.837	(0.856)	0.623	(0.817)	0.802	(0.832)
ZINC (50%)	2.111	(0.882)	1.874	(0.885)	1.174	(0.878)	1.661	(0.883)
ZINC (10%)	1.290	(0.890)	1.227	(0.896)	0.738	(0.876)	1.193	(0.892)
ZINC (1%)	0.765	(0.852)	0.845	(0.902)	0.530	(0.889)	0.807	(0.903)
(c) Hydrogen Bonding (*↑*)								
CVAE	1.574	(0.918)	0.819	(0.907)	0.240	(0.909)	0.567	(0.909)
GVAE	**4.930**	(0.902)	**3.412**	(0.901)	**2.114**	(0.901)	**2.489**	(0.935)
REINVENT	2.906	(0.829)	1.915	(0.831)	1.155	(0.843)	1.964	(0.833)
Train (50%)	3.904	(0.727)	1.153	(0.848)	0.283	(0.852)	1.203	(0.849)
Train (10%)	5.071	(0.736)	1.883	(0.860)	0.928	(0.870)	2.061	(0.863)
Train (1%)	6.157	(0.734)	2.967	(0.843)	1.962	(0.852)	3.213	(0.784)
ZINC (50%)	1.888	(0.880)	1.609	(0.882)	0.750	(0.883)	1.780	(0.881)
ZINC (10%)	2.985	(0.882)	2.538	(0.886)	1.589	(0.884)	2.699	(0.885)
ZINC (1%)	4.407	(0.890)	3.815	(0.891)	2.621	(0.894)	3.817	(0.889)

a The experimental setup is the same as in Table 2.

#### Docking Score Function Task

The key task in the benchmark is Docking Score Function. We observe that CVAE and GVAE models fail to generate compounds that achieve a higher docking score compared to the mean docking score in the ZINC data set (−8.785 for 5-HT1B compared to −4.647 and −4.955 achieved by CVAE and GVAE, respectively). The REINVENT model achieves much better performance (−9.774 for 5-HT1B). However, while docking scores attained by the molecules generated by REINVENT generally outperform the mean docking in the ZINC data set and the training set, they fall short of outperforming the top 10% molecules found in ZINC (−9.894 for 5-HT1B, with the exception of ACM2). We also draw attention to the fact that the generated molecules by REINVENT are markedly less diverse than the diversity of the training set (0.506 mean Tanimoto distance compared to 0.787 in the training set).

These results suggest that generative models applied to *de novo* drug discovery might require substantial more data to generate well-binding compounds than is typically available for training. In the key Docking Score Function task, models generally fail to outperform the top 10% from the ZINC database. It should worry us that optimizing for the docking score, which seems to be a simpler optimization target than true biological binding affinity, is already challenging given realistically sized training sets (between 1,193 and 10,225 molecules).

#### Repulsion Task

Interestingly, REINVENT performs significantly worse than GVAE and CVAE on the Repulsion task. All models fail to outperform the top 10% found in the ZINC data set. We observe markedly lower diversity of molecules generated by REINVENT compared to the training set.

#### Hydrogen Bonding Task

The Hydrogen Bonding task is the simplest, and both GVAE and REINVENT generate molecules that almost match the top 1% molecules found in the ZINC database and the training set. We again observe relatively low diversity of molecules generated by REINVENT.

#### Generated Molecules

**Figure 3:**
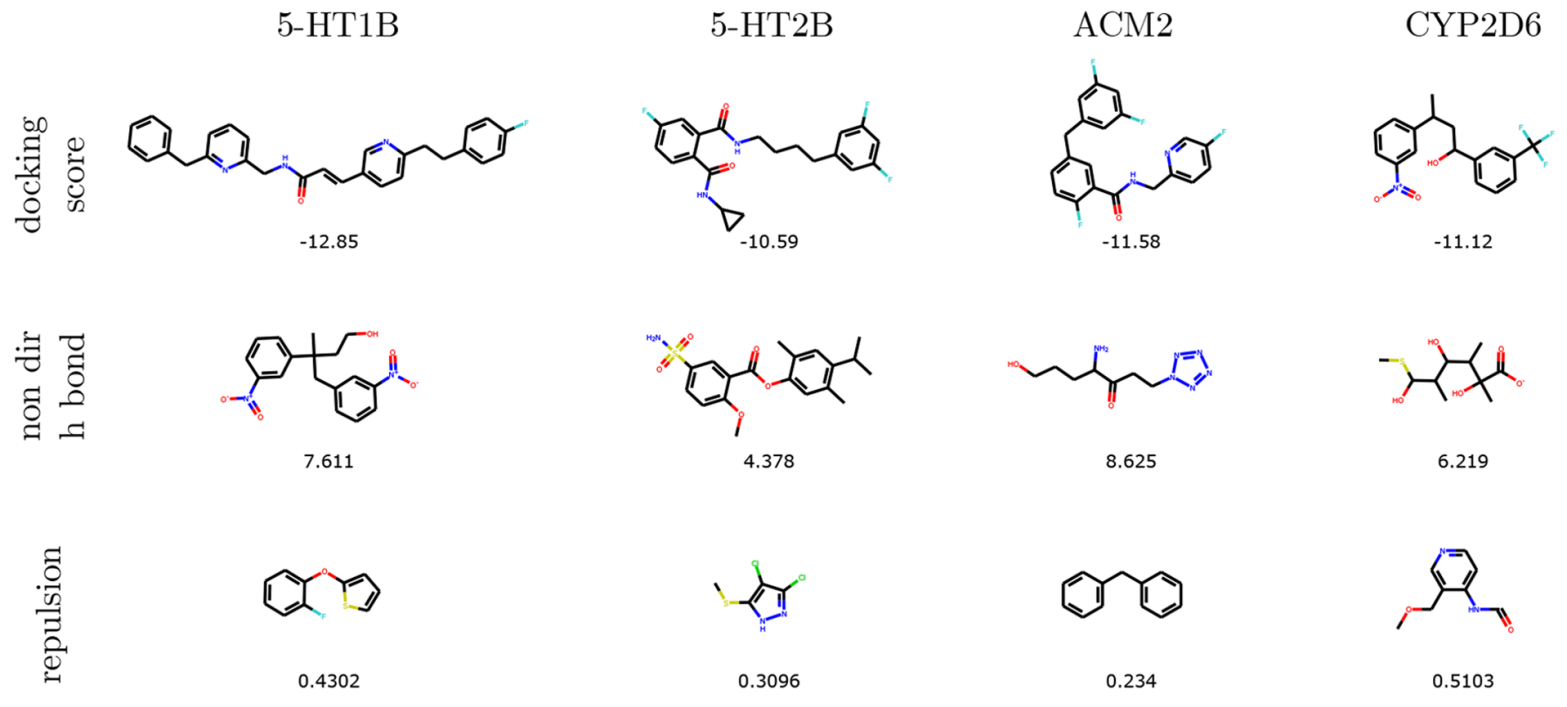
Best scoring molecules generated by REINVENT in each of the three tasks composing the benchmark.

Figure 3 shows the best scoring molecules generated by REINVENT. We observe that optimizing each objective promotes different structural motifs. For example, the best scoring molecules in the Repulsion task are small, which intuitively enables them to easily fit into the binding pocket, achieving lower repulsion values than the top 1% molecules in the training set.

**Figure 4:**
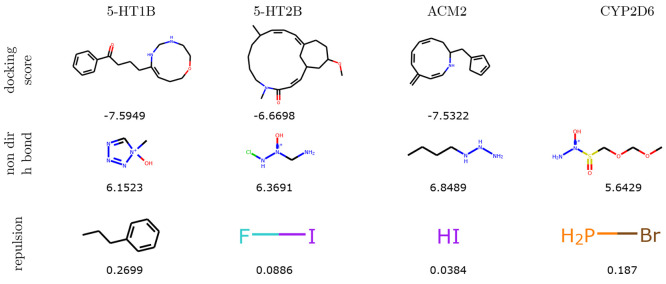
Best scoring molecules generated by CVAE in each of the three tasks composing the benchmark. Missing compounds correspond to the failed optimizations.

**Figure 5:**
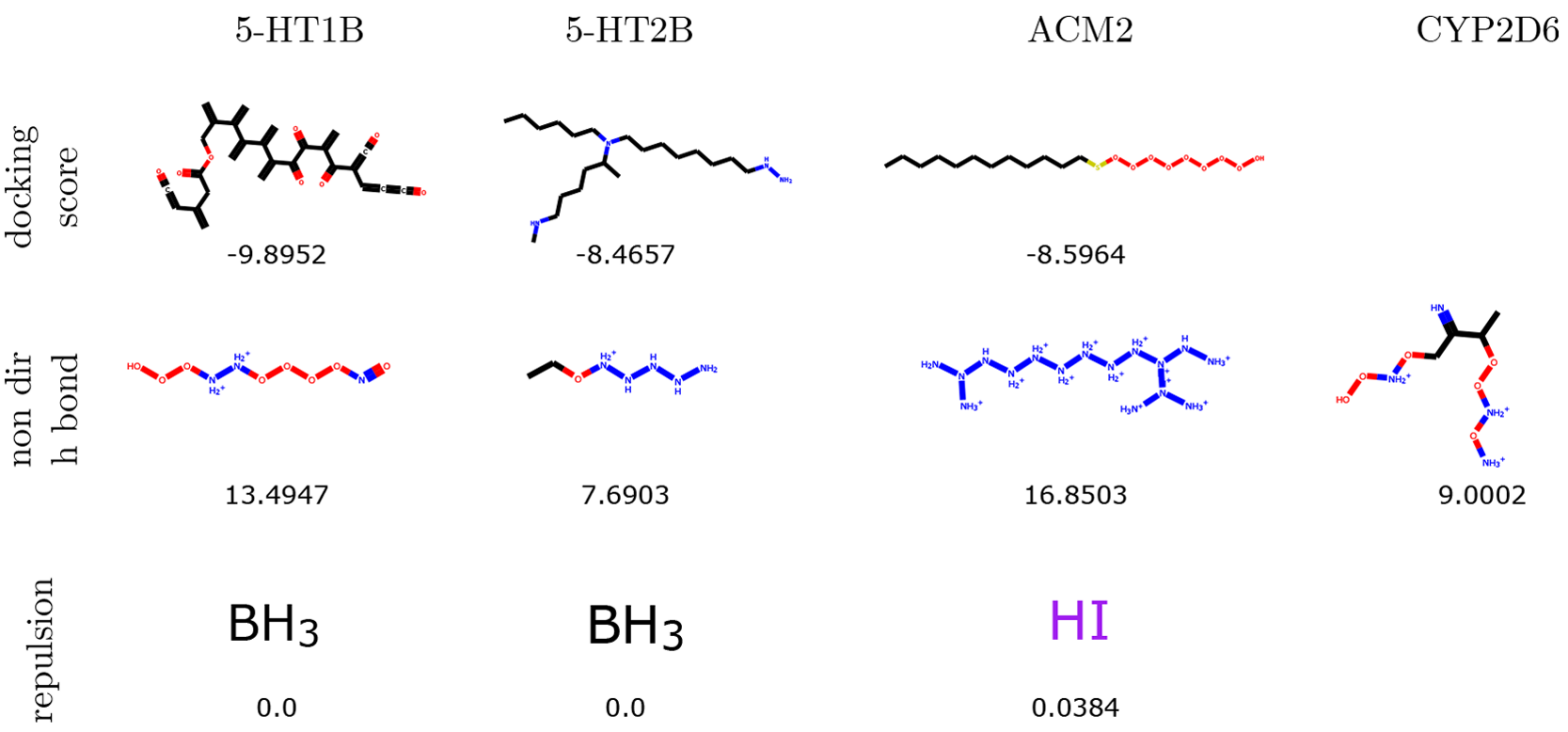
Best scoring molecules generated by GVAE in each of the three tasks composing the benchmark. Missing compounds correspond to the failed optimizations.

Similarly, there are clear patterns visible in the top molecules of CVAE and GVAE (Figures 4 and 5). For example, CVAE generates macrocycles in the task of docking score optimization, while GVAE generates long chains with no cycles when optimizing the same objective. These models also create oxygen or nitrogen chains when optimizing Hydrogen Bonding, and very small molecules (often less than 3 heavy atoms) for the Repulsion task.

**Figure 6:**
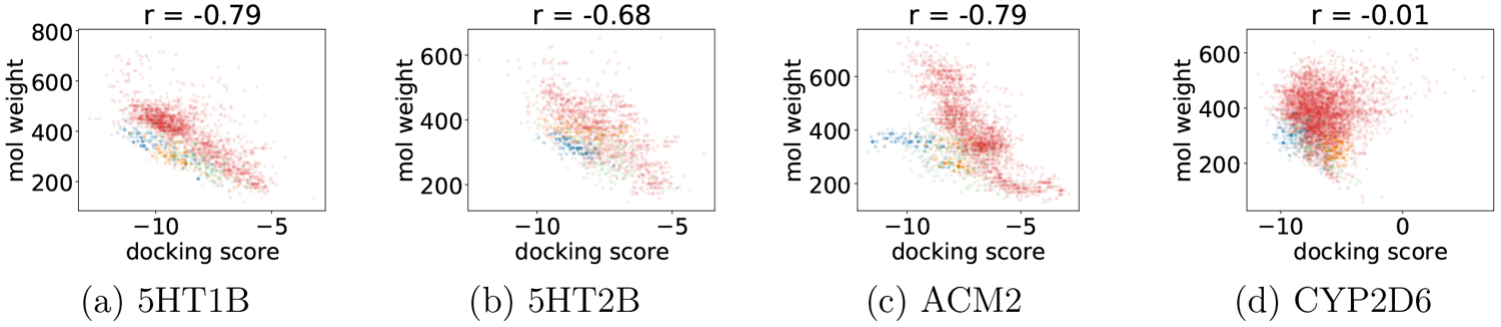
Correlation between docking score and molecular weight. The training set is marked with red dots, and the compounds generated by REINVENT by enhancing different optimization targets are colored in blue (Docking Score Function), orange (Hydrogen Bonding), and green (Repulsion).

**Figure 7:**
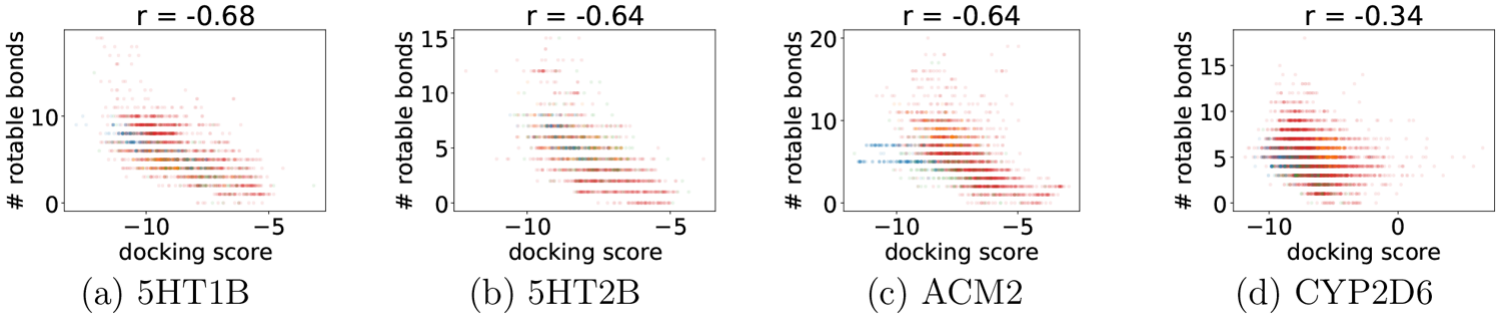
Correlation between docking score and the number of rotatable bonds. The training set is marked with red dots, and the compounds generated by REINVENT by enhancing different optimization targets are colored in blue (Docking Score Function), orange (Hydrogen Bonding), and green (Repulsion).

We noticed a moderately strong correlation between docking scores and the number of rotatable bonds or molecular weight. Figures 6 and 7 show that, with the increasing number of rotatable bonds or molecular weight, the docking scores improve. For the number of rotatable bonds, the generated compounds are well mixed with the training data marginal distribution. On the other hand, the distribution of generated compounds is shifted toward better docking scores and smaller molecular weights in the case of the weight-to-docking-score relation. In other words, molecules achieve better docking scores at the same molecular weight after the optimization. The correlations are weaker for CYP2D6, which may be caused by a bigger binding site of this enzyme. However, the last observation about molecular weights holds.

From the chemical point of view, REINVENT produced the most consistent ligands with the highest possibility of desired biological activity. When different optimization approaches are considered, the best results were produced during the docking score optimization. Nondir h-bond optimization produced compounds with sometimes a high number of moieties able to produce a hydrogen bond. In the repulsion task, the produced compounds are correct from the chemical point of view. The drug-likeliness of compounds produced by CVAE and GVAE is lower (although they still meet criteria included in the Lipiski Rule of Five), but they still can be used in the docking benchmark task. The poor quality of the generated compounds is not surprising, as this issue was previously observed for other unrestricted *de novo* generative models.^36^

## Conclusion

As concluded by Coley et al.,^10^ “the current evaluations for generative models do not reflect the complexity of real discovery problems”. Motivated by this, we proposed a new, more realistic, benchmark tailored to *de novo* drug design, using docking score as the optimization target. Code to evaluate new models is available at https://github.com/cieplinski-tobiasz/smina-docking-benchmark.

Our results suggest that generative models applied to *de novo* drug discovery pipelines might require substantially more data to generate realistic compounds than is typically available for training. Despite using over 1,000 compounds for training (between 1,074 and 3,780), the best docking scores generally do not outperform the top 10% docking scores in the ZINC data set. The docking score is only a simple proxy of the actual binding affinity, and as such, it should worry us that it is already challenging to optimize.

On a more optimistic note, the tested models achieved much better performance on the simplest task in the benchmark, which is to optimize a single term in the SMINA scoring function involving the number of hydrogen bonds to the binding site. This suggests that producing compounds that optimize the docking score based on the provided data set is an attainable, albeit challenging, task. We hope our benchmark better reflects the complexity of real discovery problems and will serve as a stepping stone toward developing better *de novo* models for drug discovery.

## Data Availability

The data and code used in this project are available at https://github.com/cieplinski-tobiasz/smina-docking-benchmark.

## Supporting Information Available

Default SMINA scoring function, Vinardo scoring function, model details (including VAE and RF hyperparameters), and data set details (PDF)
