# Supplementary material for: Generative Models Should at Least Be Able to Design Molecules That Dock Well: A New Benchmark
Source: J Chem Inf Model. 2023 May 24;63(11):3238–47. doi: 10.1021/acs.jcim.2c01355 (PMC10268949; doi:10.1021/acs.jcim.2c01355)
Supplement: Supplementary file 1 — ci2c01355_si_001.pdf [file ci2c01355_si_001.pdf]

Supporting Information

Generative models should at least be able to design molecules that dock well: a new benchmark

Tobiasz Ciepliński,[†] Tomasz Danel,[†] Sabina Podlewska,[‡] and Stanisław Jastrzębski[*,¶,†]

†*Faculty of Mathematics and Computer Science, Jagiellonian University, Łojasiewicza 6, 30-348 Kraków, Poland*

‡*Maj Institute of Pharmacology, Polish Academy of Sciences, Smętna 12, 31-343 Kraków, Poland*

¶*Molecule.one, Al. Jerozolimskie 96, 00-807 Warsaw, Poland*

E-mail: staszek.jastrzebski@gmail.com

Default SMINA scoring function

We include the definitions of SMINA's default scoring function components and weights used for calculating docking score in score only mode. a_1 and a_2 denote atoms, $d(a_1, a_2)$ is the distance between atoms, d_{opt} is the sum of their van der Waals radii and $d_{\text{diff}}(a_1, a_2) = d(a_1, a_2) - d_{\text{opt}}(a_1, a_2)$. Distance unit is Angstrom (10^{-10}m).

$$
\begin{aligned}
\text{Vina docking score} = & - 0.035579 \cdot \text{gauss}(o = 0, w = 0.5) \\
& - 0.005156 \cdot \text{gauss}(o = 3, w = 2) \\
& + 0.840245 \cdot \text{repulsion (o=0, c=8)} \\
& - 0.035069 \cdot \text{hydrophobic (g=0.5, b=1.5, c=8)} \\
& - 0.587439 \cdot \text{non\_dir\_h\_bond (g=-0.7, b=0, c=8)}
\end{aligned}
$$

$$
\text{gauss}(a_1, a_2) = \exp\left(-\left(\frac{d_{\text{diff}}(a_1, a_2) - o}{w}\right)^2\right)
$$

$$
\text{repulsion}(a_1, a_2) = \begin{cases} (d_{\text{diff}}(a_1, a_2) - o)^2, & d_{\text{diff}}(a_1, a_2) - o < 0 \\ 0, & \text{otherwise} \end{cases}
$$

$$
\text{hydrophobic}(a_1, a_2) = \begin{cases} 0, & a_1 \text{ is not hydrophobic or } a_2 \text{ is not hydrophobic} \\ 1, & d_{\text{diff}}(a_1, a_2) < g \\ 0, & d_{\text{diff}}(a_1, a_2) \geq b \\ \frac{d_{\text{diff}}(a_1, a_2) - b}{g - b}, & \text{otherwise} \end{cases}
$$

$$
\text{non\_dir\_h\_bond}(a_1, a_2) = \begin{cases} 0, & (a_1, a_2) \text{ do not form hydrogen bond} \\ 1, & d_{\text{diff}}(a_1, a_2) < g \\ 0, & d_{\text{diff}}(a_1, a_2) \geq b \\ \frac{d_{\text{diff}}(a_1, a_2) - b}{g - b}, & \text{otherwise} \end{cases}
$$

Vinardo scoring function

The Vinardo scoring function used in our benchmark is a docking score derived from the default SMINA docking score. It uses the same components, but with different weights and

parameters.

$$\begin{aligned}\text{Docking score} =& -0.045 \cdot \text{gauss}(o=0, w=0.8, c=8)\\ &+ 0.8 \cdot \text{repulsion(o=0, c=8)}\\ &- 0.035 \cdot \text{hydrophobic(g=0.0, b=2.5, c=8)}\\ &- 0.6 \cdot \text{non\_dir\_h\_bond(g=-0.6, b=0, c=8)},\end{aligned}$$

Model details

We include hyperparameters and training settings used in our models. Our code is available at https://github.com/cieplinski-tobiasz/smina-docking-benchmark.

MLP is used to predict docking score from CVAE or GVAE latent space representation of molecule. It is a simple feed forward neural network with one hidden layer. Hyperparameters of this model are listed in Table S1.

Table S1: MLP hyperparameters

	Parameter
Training epochs	50
Layers number	1
Hidden layer dim	1000
Loss function	Mean Squared Error
Optimizer	Adam
Learning rate	0.001

Both Chemical VAE and Grammar VAE are based on variational autoencoder model with stacked convolution layers in its encoder part and stacked GRU layers in decoder part. What differs them is the way that SMILES is encoded to one hot vector. Chemical VAE encodes each character of SMILES to separate one-hot vector, while Grammar VAE forms a parse tree from SMILES and encodes the parse rules. Details for CVAE are listed in Table S2 and for GVAE in Table S3.

The REINVENT model was used with the default parameters of the original implemen-

Table S2: Chemical VAE hyperparameters

	Parameter
MLP learning rate	0.05
MLP descent iterations	50
Fine-tuning batch size	256
Fine-tuning epochs	5
Latent space dim	196
Encoder convolution layers number	4
Decoder GRU layers number	4

Table S3: Grammar VAE hyperparameters

	Parameter
MLP learning rate	0.01
MLP descent iterations	50
Fine-tuning batch size	256
Fine-tuning epochs	5
Latent space dim	56
Encoder convolution layers number	3
Decoder GRU layers number	3

tation provided by Olivecrona et al. The model consists of 3 GRU layers and was pretrained on the ChEMBL dataset. The RL agent was trained for 200 steps with a random forest scoring function. The hyperparameters of the random forest were chosen by a grid search, and the resulting configuration is shown in Table S4.

Table S4: RF hyperparameters

	Parameter
number of estimators	500
criterion	gini
maximum number of features in splits	sqrt(number of features)

Dataset details

The compound sets were downloaded from the ChEMBL database. All records referring to human- and rat-based records were taken into account. Compounds with Ki values below 100 nM (referred to as active compounds) and above 1000 nM (inactive ones) were taken

into account. Only binding data were considered, and it was assumed that IC50 = Ki/2. The compound protonation states were generated for pH = 7.4.

The crystal structures for docking were fetched from the PDB database, the following structures were used in the study: 4IAQ for 5-HT1B, 4NC3 for 4-HT2B, 3UON for ACM2, 3QM4 for CYP3D6, 4BVN for ADRB1, 5C1M for MOR, 5IU4 for A2A, and 6CM4 for D2. The Protein Preparation Wizard from the Schrödinger molecular modeling package was used for protein preparation for docking.
